# Successful prehospital ECMO in drowning resuscitation after prolonged submersion

**DOI:** 10.1016/j.resplu.2024.100685

**Published:** 2024-06-07

**Authors:** Jeroen Seesink, Wietske van der Wielen, Dinis Dos Reis Miranda, Xavier J.R. Moors

**Affiliations:** a Department of Anaesthesiology, Erasmus MC University Medical Centre, Rotterdam, The Netherlands; b Amsterdam University Medical Centre, Amsterdam, The Netherlands; c Helicopter Emergency Medical Services, Trauma Centre Zuid-West Nederland, Erasmus MC University Medical Centre, Rotterdam, The Netherlands; d Department of Intensive Care, Erasmus MC University Medical Centre, Rotterdam, The Netherlands

**Keywords:** Cardiopulmonary resuscitation (CPR), Drowning Resuscitation, Drowning, Extracorporeal membrane oxygenation (ECMO), Helicopter Emergency Medical Service (HEMS), Out-of-Hospital Cardiac Arrest (OHCA), Prehospital veno-arterial extracorporeal membrane oxygenation (VA-ECMO)

## Abstract

An 18-year-old drowning victim was successfully resuscitated using prehospital veno-arterial extracorporeal membrane oxygenation (VA-ECMO). Despite 24 min of submersion in water with a surface temperature of 15 °C, the patient was cannulated on-scene and transported to a trauma center. After ICU admission on VA-ECMO, he was decannulated and extubated by day 5. He was transferred to a peripheral hospital on day 6 and discharged home after 3.5 weeks with favorable neurological outcome of a Cerebral Performance Categories (CPC) score of 1 out of 5. This case underscores the potential of prehospital ECMO in drowning cases within a well-equipped emergency response system.

## Introduction

Globally, approximately 236,000 individuals drown annually. This accounts for roughly 7% of all injury-related deaths.^1^ In the Netherlands, there are approximately 275 fatal drownings per year, with a rising trend observed in recent years.^2^ The mortality rate associated with prolonged submersion (≥15–25 min) in drowning is high.^3^ Clinical studies have demonstrated the benefits of extracorporeal cardiopulmonary resuscitation (ECPR) in cases of out-of-hospital cardiac arrest (OHCA), over standard advanced cardiac life support (ACLS) in selected patients, as ECPR facilitates immediate hemodynamic and respiratory stabilization of patients experiencing refractory cardiac arrest.^4^, ^5^, ^6^, ^7^ Therefore, guidelines recommend considering ECPR in cases of OHCA where conventional cardiopulmonary resuscitation (CPR) fails to restore spontaneous circulation, provided that the necessary infrastructure is available for its implementation.^8^ This recommendation extends to OHCA related to drowning.^9^, ^10^ It is possible that survival outcomes in OHCA could be further improved by implementing ECPR prehospitally.^6^, ^7^, ^11^ However, to our knowledge, on-scene ECPR has not yet been applied specifically in cases of confirmed prolonged submersion.

## Methods

The authors obtained written informed consent from the patient, who was competent to sign for himself, following our local institutional protocol. All data were de-identified and reviewed securely.

## Case report

**Fig. 1:**
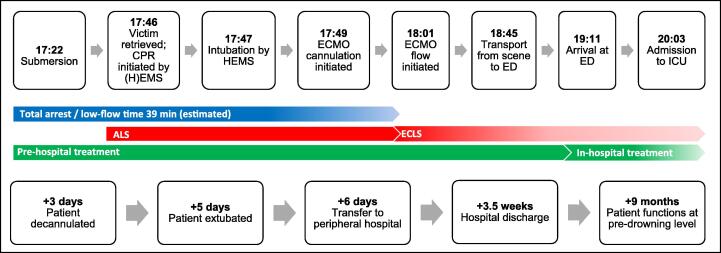
Timeline.

On a summer day with a temperature of 27.8 °C according to data from the Royal Netherlands Meteorological Institute (KNMI), a previously healthy 18-year-old male experiences distress while swimming in a recreational pond, in the presence of bystanders and lifeguards. Due to fatigue and subsequent panicking, the man submerges underwater at 17:22 (Fig. 1.). After a documented submersion time of 24 min, the victim is retrieved from the water by rescue divers from 8 m depth in water of 15 °C at the surface (KNMI). The ambulance and Helicopter Emergency Medical Service (HEMS) are prepared on the beach to initiate immediate resuscitation. The initial rhythm observed is fine ventricular fibrillation (VF), for which a single shock of 200 J is delivered. Mechanical chest compressions are initiated, and endotracheal intubation is performed by the HEMS physician, encountering significant regurgitation and fluid emanating from the mouth. Subsequently, ventilation with 10 cmH_2_O Positive End-Expiratory Pressure (PEEP) is initiated, and the HEMS team begins cannulation for Veno-Arterial Extracorporeal Membrane Oxygenation (VA-ECMO) on site. The right femoral artery and left femoral vein were punctured for guidewire placement, followed by the insertion of a 25Fr venous catheter and a 17Fr arterial catheter. Meanwhile, five cycles of CPR have elapsed, during which there was persistent pulseless electrical activity based on bradycardia with no output. By this time, 1 mg of epinephrine has been administered intravenously twice. After canulation, VA-ECMO was initiated, and manual chest compressions could be discontinued. Bilateral rhonchi can be heard during auscultation. The first mean arterial pressure (MAP) measured in the arterial line is 85 mmHg during a broad complex rhythm. The core temperature at that moment is 30.5 °C.

The patient is transported to an academic level-1 trauma center. During transport, the QRS complexes narrow, the heart rhythm stabilizes, the MAP increases to 110 mmHg and the patient begins to breathe spontaneously. Intravenous fluids and sedation are administered. After arriving at the emergency room, the pupils are equal and reactive to light bilaterally. By now, the temperature is 32.4 °C. The calculated Hypothermia Outcome Prediction after ECLS (HOPE) score showed a 5% probability of survival to hospital discharge.^12^ A CT scan of the abdomen and thorax reveals no other injuries besides extensive consolidations in the dorsal lung fields and bilateral pulmonary edema. The ECG shows sinus rhythm with nonspecific intraventricular conduction delay characterized by a QRS duration of 110 ms and Osborn waves. A quick look transthoracic echocardiography (TTE) reveals no pericardial effusion, no right ventricular overload. Based on the description of the incident, there was no indication for toxicology screening.

The patient is admitted to the Intensive Care Unit (ICU) and undergoes 24 h of targeted temperature management with cooling to 33 °C. The patient is mechanically ventilated and the VA-ECMO settings are as follows: 3.7 L/min flow at 3500 rpm, sweep flow 2.5L with FiO_2_ at 60%. He is pharmacologically supported with norepinephrine and milrinone. The arterial blood gas on admission is as follows: pH 7.08, pCO_2_ 5.8 kPa, pO_2_ 26.1 kPa, standard bicarbonate 12.3 mmol/L, base excess −17 mmol/L, and lactate 14.7 mmol/L. Heparin is initiated at 30,000 IU/day guided by the activated partial thromboplastin time. Immediately upon ICU admission, an arterial retrograde leg cannula is placed in the right leg during surgery. TTE now shows a globally reduced systolic left ventricular function without significant valvular abnormalities and good right ventricular function. After 24 h, the patient is warmed at a rate of 0.25 °C per hour. Hemodynamic and respiratory support can be gradually reduced.

Longitudinal Electroencephalography Mapping (LEM) examination after 24 h reveals a consistently evolving background pattern. After 2 days, the patient is neurologically scored as E1M4Vt, with intact brainstem reflexes. VA-ECMO support is successfully reduced and can be removed 3 days after admission, and inotropy can be discontinued. The ventilation conditions can be smoothly reduced, and his neurology rapidly improved to bilateral M6. The patient is extubated on day five after admission and transferred to a peripheral hospital on day six. At that time, there is complete neurological recovery except for some bradyphrenia, but this could be due to a language barrier. Three and a half weeks later, he can be discharged home in good health with a favorable neurological outcome CPC score of 1 out of 5. Three months after the accident, he completed his rehabilitation program, and after 8 months, he resumed his Dutch language studies. Now he is functioning nearly as he did before the incident, apart from mild impairment in memory, which was identified and confirmed during outpatient follow-up at the hospital.

## Discussion

Drowning has an asphyxial nature with associated cerebral hypoxemia and cardiac arrest, leading to high mortality among victims.^13^ The likelihood of survival with a favorable outcome is extremely unlikely in cases of prolonged submersion (≥15-25 min).^3^, ^13^, ^14^

Some literature supports that drowning in cold water(≤6°C) increases the likelihood of survival with a favorable neurological outcome, especially if the individual has undergone hypothermia prior to cardiac arrest.^13^, ^14^ This is highlighted by the singular reported case in which prehospital ECMO was administered to a drowning victim with a positive neurological outcome, where the water temperature was 2 °C and there was possibly only a brief duration of submersion.^15^ With a water surface temperature of 15 °C, it is unlikely that in our case there was hypothermia prior to submersion, and therefore the chance of survival with a favorable outcome was extremely small. However, local divers and divers from the fire brigade have estimated the temperature at 8 m depth to be 8 to 10 °C, which is significantly lower than at the surface.

Hypothermia, if not treated properly, can lead to arrhythmias and cardiac arrest. Therefore, drowned patients frequently require both rewarming and cardiopulmonary support.^5^, ^13^, ^14^ In the in-hospital setting, extracorporeal life support (ECLS) is the preferred rewarming technique for patients with hypothermic cardiac arrest. ECMO has been reported in clinical trials and observational studies to improve survival rates with favorable neurological outcomes, compared to standard ACLS measures.^4^ The International Liaison Committee on Resuscitation (ILCOR) guidelines currently advocate for the use of VA-ECMO as a resuscitation method in cases of refractory OHCA. This recommendation includes cases where the cause of cardiac arrest is drowning or hypothermia.^8^ ECLS effectively combines rewarming with adequate blood supply and oxygenation of the brain and heart. Although survival rates and rates of favorable neurological outcome after ECMO treatment vary between studies, in-hospital ECLS has shown promise as a treatment for cardiac arrest due to hypothermia and drowning, resulting in favorable neurological outcomes in selected patients or serve as a bridge-to-decision tool.^7^, ^14^, ^15^, ^16^, ^17^ The most reliable tool for predicting survival in hypothermic drowning victims is the HOPE score.^12^, ^18^ Remarkably, in this case, survival with a favorable neurological outcome occurred at a HOPE score of only 5%.

Timely CPR and minimal low-flow time enhance the likelihood of survival after cardiac arrest, and an important prognostic factor associated with good outcome after ECPR is shorter time to cannulation. Early application of ECMO, with blood flow starting within 60 min, is crucial for enabling this advanced treatment with the possibility of a good outcome.^4^, ^6^, ^7^, ^14^ However, transferring a patient from the scene of an OHCA while maintaining ongoing CPR within one hour presents a challenging task. Cannulation by a HEMS team in a prehospital setting could significantly reduce low-flow times in carefully selected patients.^6^, ^7^, ^11^, ^19^ It should, however, be considered that ECPR is not a riskless procedure, with access site complications occurring in approximately 10%, and major bleeding in 5–10% of in-hospital ECPR.^20^ In addition to this, ECPR represents a resource-intensive and costly procedure.^6^, ^7^

## Conclusion

Prehospital ECPR was performed successfully in a drowned patient with cardiac arrest. The survival with favorable neurological outcomes, despite extremely unfavorable circumstances, demonstrates the potential of prehospital ECMO treatment in cases of drowning within a well-equipped and trained emergency response system. This highlights the importance of further research on prehospital ECPR and its feasibility for standard practice.

## CRediT authorship contribution statement

**Jeroen Seesink:** Writing – original draft, Visualization, Supervision, Resources, Project administration, Methodology, Investigation, Conceptualization. **Wietske van der Wielen:** Writing – original draft, Visualization, Methodology, Investigation, Conceptualization. **Dinis Dos Reis Miranda:** Writing – review & editing, Supervision, Resources, Methodology, Investigation, Conceptualization. **Xavier J.R. Moors:** Writing – review & editing, Writing – original draft, Supervision, Resources, Methodology, Investigation, Conceptualization.

## Declaration of competing interest

The authors declare that they have no known competing financial interests or personal relationships that could have appeared to influence the work reported in this paper.
